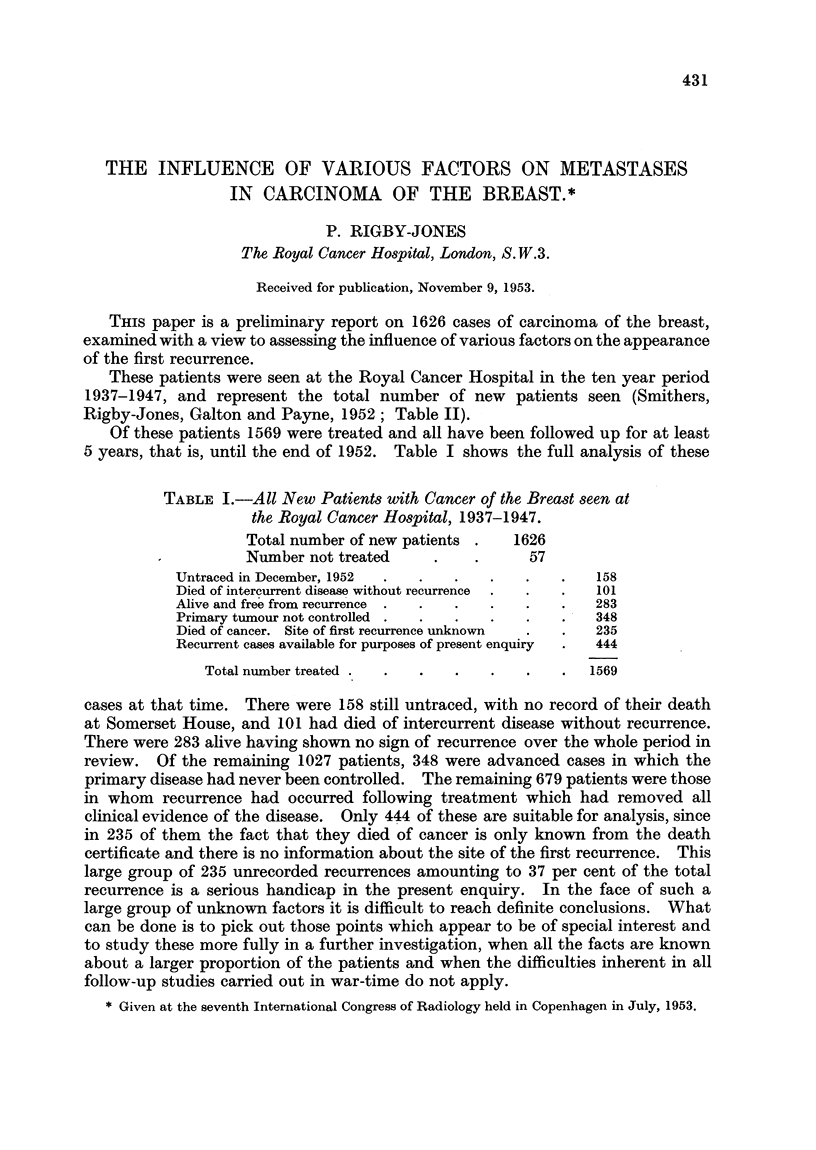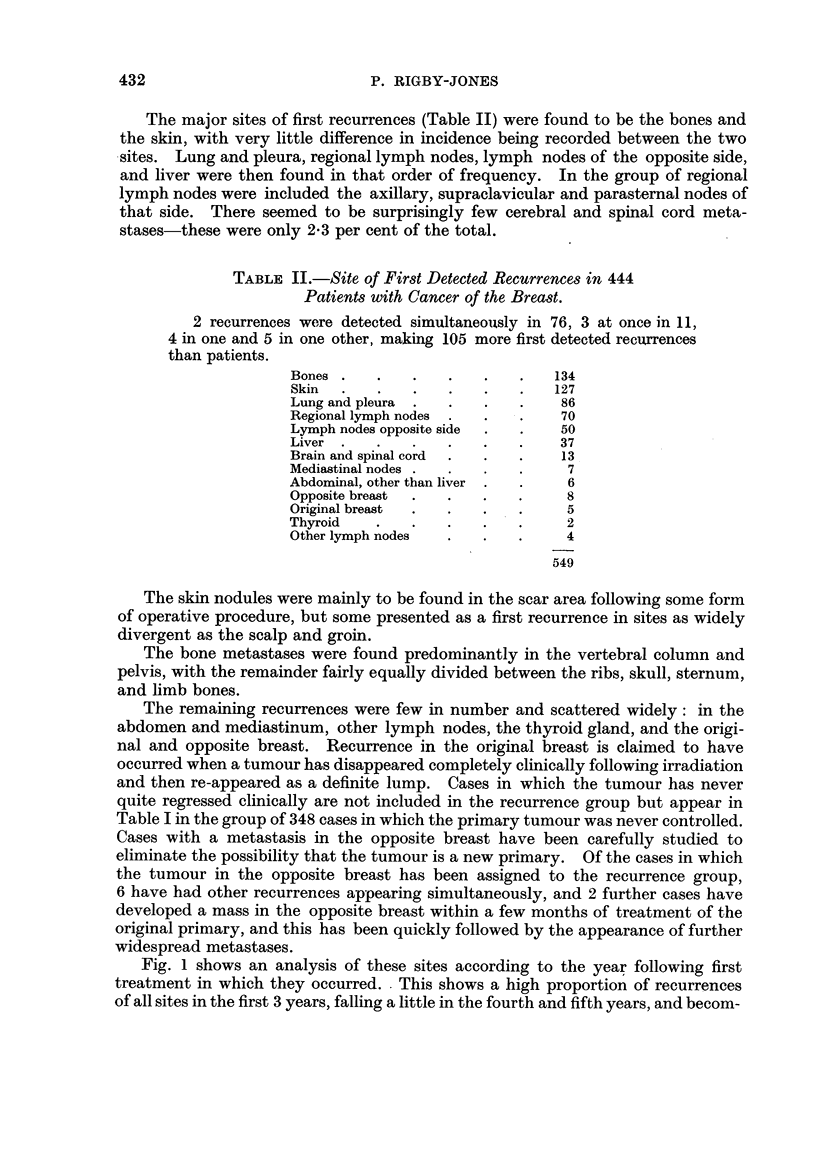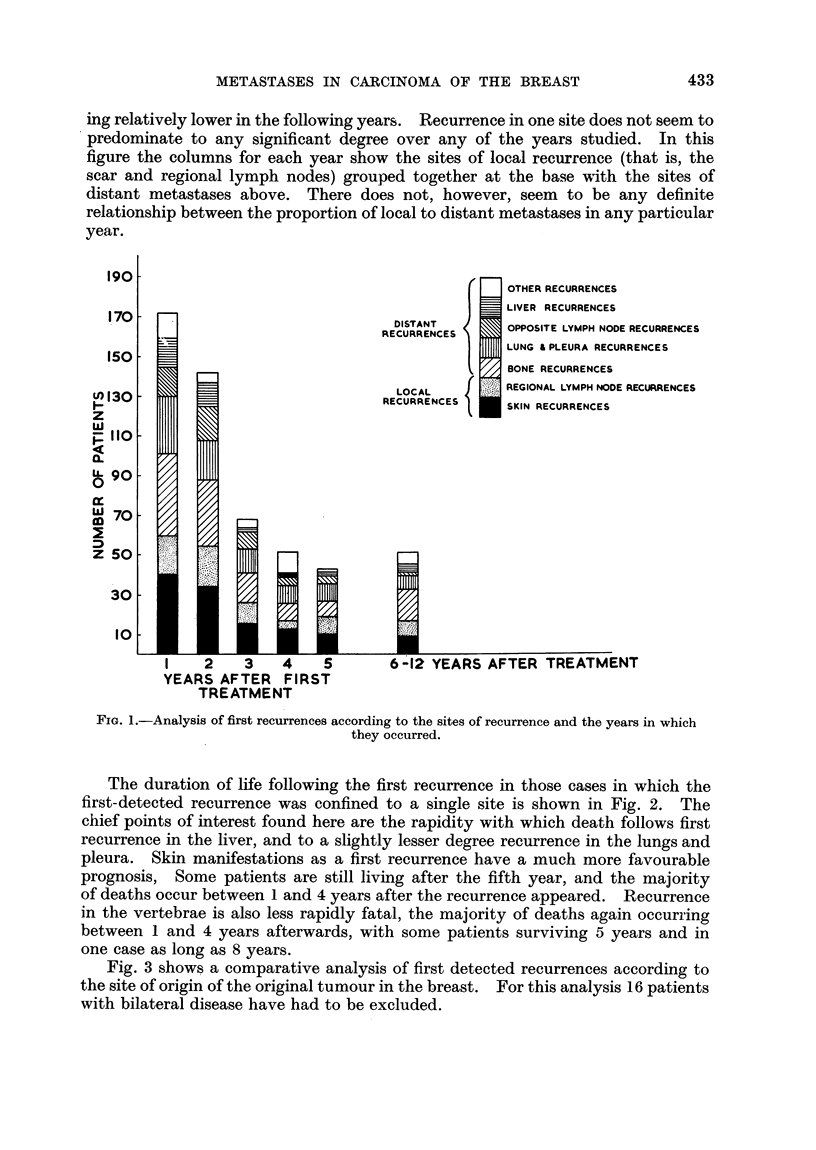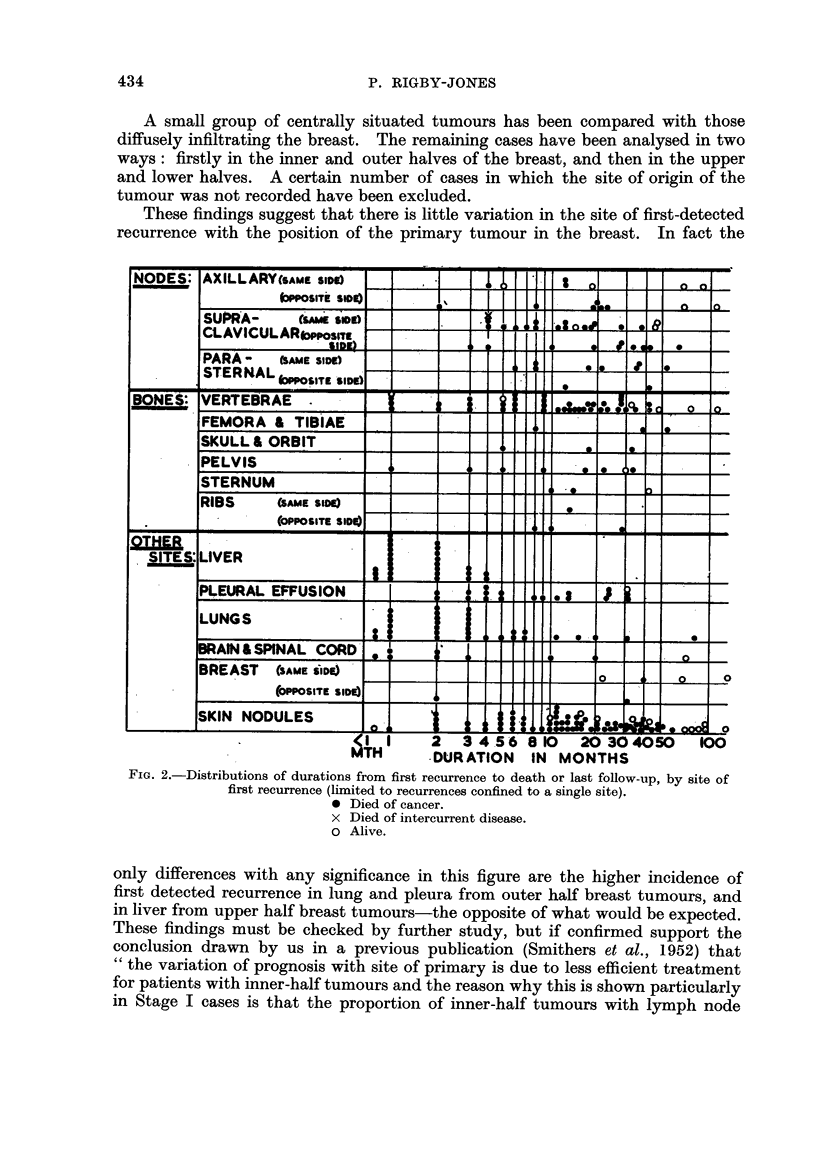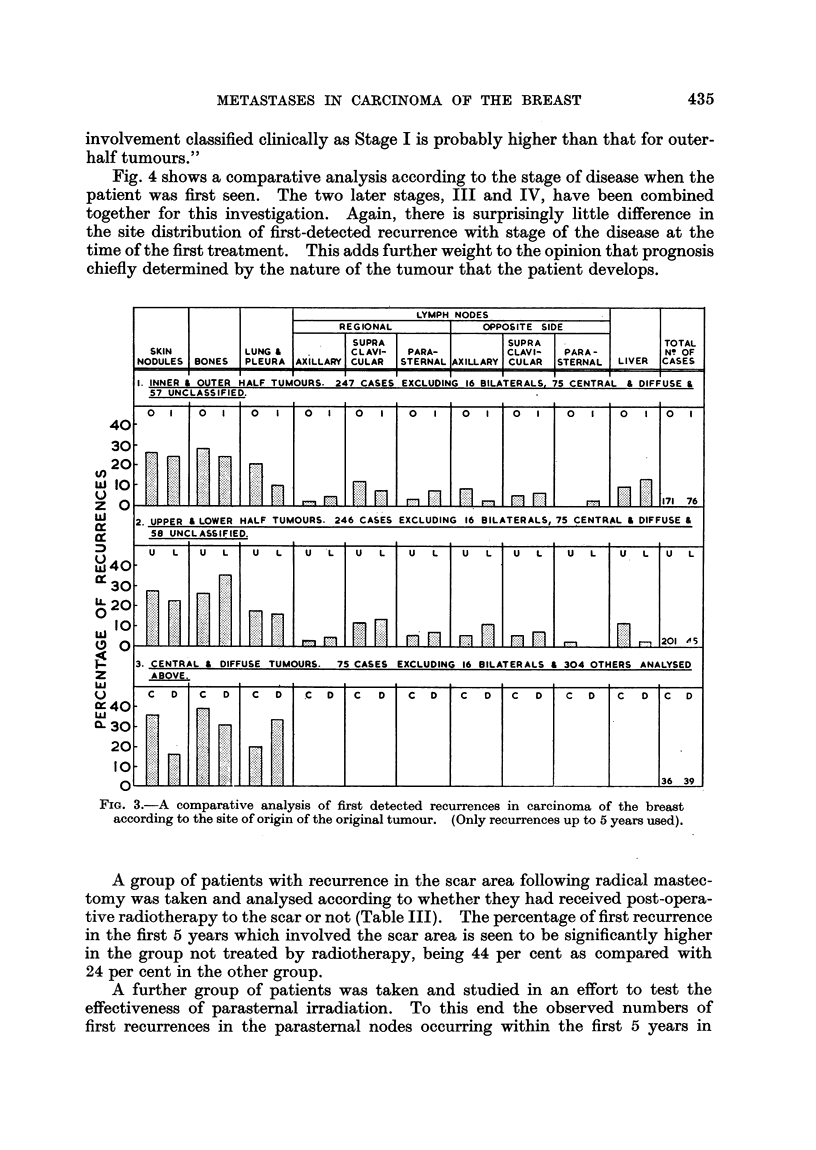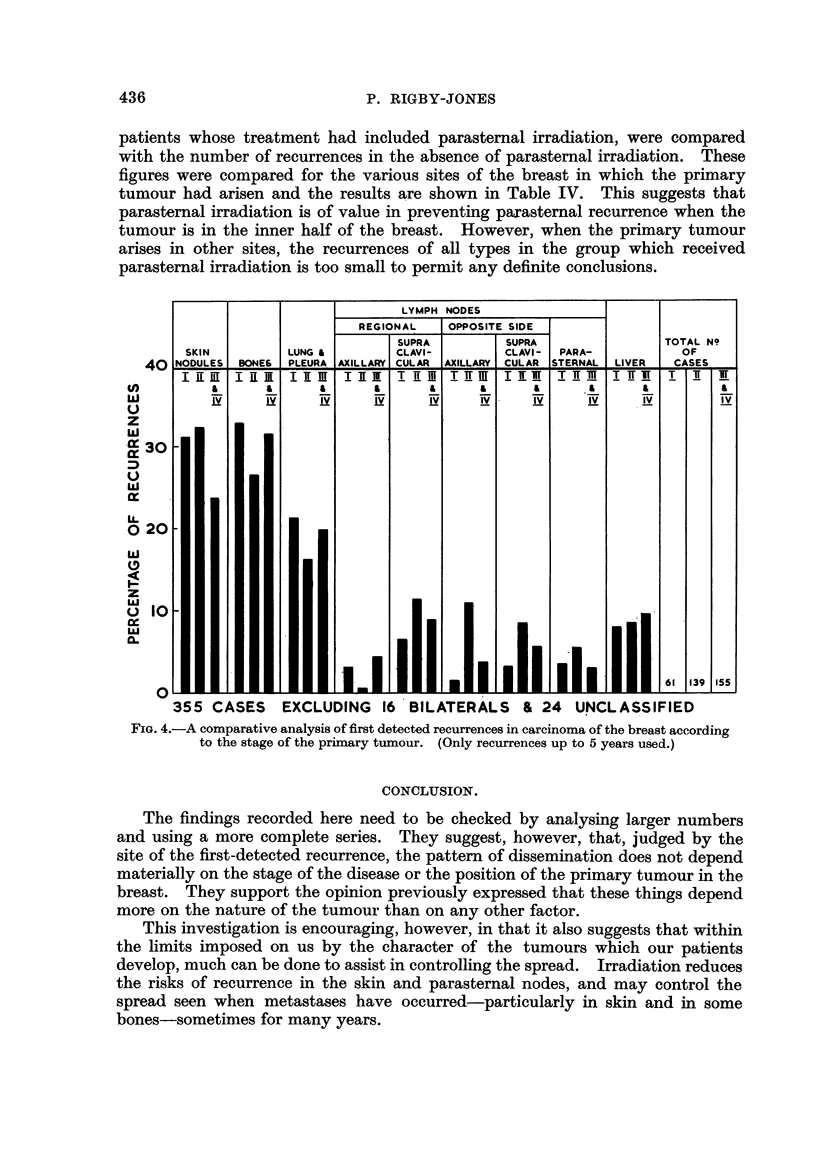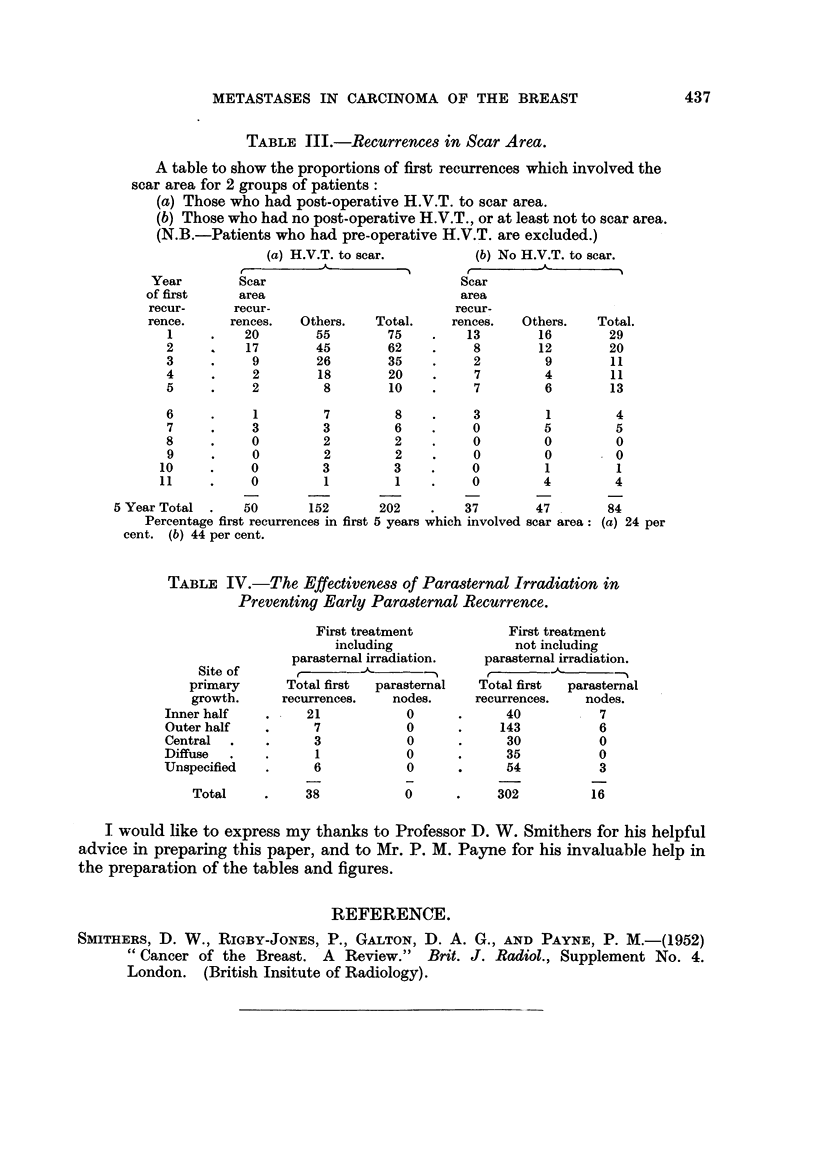# The Influence of Various Factors on Metastases in Carcinoma of the Breast*

**DOI:** 10.1038/bjc.1953.43

**Published:** 1953-12

**Authors:** P. Rigby-Jones

## Abstract

**Images:**


### THE INFLUENCE OF VARIOUS FACTORS ON METASTASES IN CARCINOMA OF THE BREAST.*

P. RIGBY-JONES

*The Royal Cancer Hospital, London, S.W.3.*

Received for publication, November 9, 1953.

THIS paper is a preliminary report on 1626 cases of carcinoma of the breast, examined with a view to assessing the influence of various factors on the appearance of the first recurrence.

These patients were seen at the Royal Cancer Hospital in the ten year period 1937–1947, and represent the total number of new patients seen (Smithers, Rigby-Jones, Galton and Payne, 1952 ; Table II).

Of these patients 1569 were treated and all have been followed up for at least 5 years, that is, until the end of 1952. Table I shows the full analysis of these

TABLE I.—*All New Patients with Cancer of the Breast seen at the Royal Cancer Hospital*, 1937–1947.

| | |
|---|---|
| Total number of new patients | 1626 |
| Number not treated | 57 |
| Untraced in December, 1952 | 158 |
| Died of intercurrent disease without recurrence | 101 |
| Alive and free from recurrence | 283 |
| Primary tumour not controlled | 348 |
| Died of cancer. Site of first recurrence unknown | 235 |
| Recurrent cases available for purposes of present enquiry | 444 |
| Total number treated | 1569 |

cases at that time. There were 158 still untraced, with no record of their death at Somerset House, and 101 had died of intercurrent disease without recurrence. There were 283 alive having shown no sign of recurrence over the whole period in review. Of the remaining 1027 patients, 348 were advanced cases in which the primary disease had never been controlled. The remaining 679 patients were those in whom recurrence had occurred following treatment which had removed all clinical evidence of the disease. Only 444 of these are suitable for analysis, since in 235 of them the fact that they died of cancer is only known from the death certificate and there is no information about the site of the first recurrence. This large group of 235 unrecorded recurrences amounting to 37 per cent of the total recurrence is a serious handicap in the present enquiry. In the face of such a large group of unknown factors it is difficult to reach definite conclusions. What can be done is to pick out those points which appear to be of special interest and to study these more fully in a further investigation, when all the facts are known about a larger proportion of the patients and when the difficulties inherent in all follow-up studies carried out in war-time do not apply.

* Given at the seventh International Congress of Radiology held in Copenhagen in July, 1953.

The major sites of first recurrences (Table II) were found to be the bones and the skin, with very little difference in incidence being recorded between the two sites. Lung and pleura, regional lymph nodes, lymph nodes of the opposite side, and liver were then found in that order of frequency. In the group of regional lymph nodes were included the axillary, supraclavicular and parasternal nodes of that side. There seemed to be surprisingly few cerebral and spinal cord metastases—these were only 2·3 per cent of the total.

TABLE II.—*Site of First Detected Recurrences in* 444 *Patients with Cancer of the Breast.*

2 recurrences were detected simultaneously in 76, 3 at once in 11, 4 in one and 5 in one other, making 105 more first detected recurrences than patients.

| | |
|---|---|
| Bones | 134 |
| Skin | 127 |
| Lung and pleura | 86 |
| Regional lymph nodes | 70 |
| Lymph nodes opposite side | 50 |
| Liver | 37 |
| Brain and spinal cord | 13 |
| Mediastinal nodes | 7 |
| Abdominal, other than liver | 6 |
| Opposite breast | 8 |
| Original breast | 5 |
| Thyroid | 2 |
| Other lymph nodes | 4 |
| | 549 |

The skin nodules were mainly to be found in the scar area following some form of operative procedure, but some presented as a first recurrence in sites as widely divergent as the scalp and groin.

The bone metastases were found predominantly in the vertebral column and pelvis, with the remainder fairly equally divided between the ribs, skull, sternum, and limb bones.

The remaining recurrences were few in number and scattered widely: in the abdomen and mediastinum, other lymph nodes, the thyroid gland, and the original and opposite breast. Recurrence in the original breast is claimed to have occurred when a tumour has disappeared completely clinically following irradiation and then re-appeared as a definite lump. Cases in which the tumour has never quite regressed clinically are not included in the recurrence group but appear in Table I in the group of 348 cases in which the primary tumour was never controlled. Cases with a metastasis in the opposite breast have been carefully studied to eliminate the possibility that the tumour is a new primary. Of the cases in which the tumour in the opposite breast has been assigned to the recurrence group, 6 have had other recurrences appearing simultaneously, and 2 further cases have developed a mass in the opposite breast within a few months of treatment of the original primary, and this has been quickly followed by the appearance of further widespread metastases.

Fig. 1 shows an analysis of these sites according to the year following first treatment in which they occurred. This shows a high proportion of recurrences of all sites in the first 3 years, falling a little in the fourth and fifth years, and becoming relatively lower in the following years. Recurrence in one site does not seem to predominate to any significant degree over any of the years studied. In this figure the columns for each year show the sites of local recurrence (that is, the scar and regional lymph nodes) grouped together at the base with the sites of distant metastases above. There does not, however, seem to be any definite relationship between the proportion of local to distant metastases in any particular year.

FIG. 1.—Analysis of first recurrences according to the sites of recurrence and the years in which they occurred.

The duration of life following the first recurrence in those cases in which the first-detected recurrence was confined to a single site is shown in Fig. 2. The chief points of interest found here are the rapidity with which death follows first recurrence in the liver, and to a slightly lesser degree recurrence in the lungs and pleura. Skin manifestations as a first recurrence have a much more favourable prognosis, Some patients are still living after the fifth year, and the majority of deaths occur between 1 and 4 years after the recurrence appeared. Recurrence in the vertebrae is also less rapidly fatal, the majority of deaths again occurring between 1 and 4 years afterwards, with some patients surviving 5 years and in one case as long as 8 years.

Fig. 3 shows a comparative analysis of first detected recurrences according to the site of origin of the original tumour in the breast. For this analysis 16 patients with bilateral disease have had to be excluded.

A small group of centrally situated tumours has been compared with those diffusely infiltrating the breast. The remaining cases have been analysed in two ways: firstly in the inner and outer halves of the breast, and then in the upper and lower halves. A certain number of cases in which the site of origin of the tumour was not recorded have been excluded.

These findings suggest that there is little variation in the site of first-detected recurrence with the position of the primary tumour in the breast. In fact the

FIG. 2.—Distributions of durations from first recurrence to death or last follow-up, by site of first recurrence (limited to recurrences confined to a single site).
● Died of cancer.
× Died of intercurrent disease.
o Alive.

only differences with any significance in this figure are the higher incidence of first detected recurrence in lung and pleura from outer half breast tumours, and in liver from upper half breast tumours—the opposite of what would be expected. These findings must be checked by further study, but if confirmed support the conclusion drawn by us in a previous publication (Smithers *et al.*, 1952) that "the variation of prognosis with site of primary is due to less efficient treatment for patients with inner-half tumours and the reason why this is shown particularly in Stage I cases is that the proportion of inner-half tumours with lymph node involvement classified clinically as Stage I is probably higher than that for outer-half tumours."

Fig. 4 shows a comparative analysis according to the stage of disease when the patient was first seen. The two later stages, III and IV, have been combined together for this investigation. Again, there is surprisingly little difference in the site distribution of first-detected recurrence with stage of the disease at the time of the first treatment. This adds further weight to the opinion that prognosis chiefly determined by the nature of the tumour that the patient develops.

FIG. 3.—A comparative analysis of first detected recurrences in carcinoma of the breast according to the site of origin of the original tumour. (Only recurrences up to 5 years used).

A group of patients with recurrence in the scar area following radical mastectomy was taken and analysed according to whether they had received post-operative radiotherapy to the scar or not (Table III). The percentage of first recurrence in the first 5 years which involved the scar area is seen to be significantly higher in the group not treated by radiotherapy, being 44 per cent as compared with 24 per cent in the other group.

A further group of patients was taken and studied in an effort to test the effectiveness of parasternal irradiation. To this end the observed numbers of first recurrences in the parasternal nodes occurring within the first 5 years in patients whose treatment had included parasternal irradiation, were compared with the number of recurrences in the absence of parasternal irradiation. These figures were compared for the various sites of the breast in which the primary tumour had arisen and the results are shown in Table IV. This suggests that parasternal irradiation is of value in preventing parasternal recurrence when the tumour is in the inner half of the breast. However, when the primary tumour arises in other sites, the recurrences of all types in the group which received parasternal irradiation is too small to permit any definite conclusions.

FIG. 4.—A comparative analysis of first detected recurrences in carcinoma of the breast according to the stage of the primary tumour. (Only recurrences up to 5 years used.)

### CONCLUSION.

The findings recorded here need to be checked by analysing larger numbers and using a more complete series. They suggest, however, that, judged by the site of the first-detected recurrence, the pattern of dissemination does not depend materially on the stage of the disease or the position of the primary tumour in the breast. They support the opinion previously expressed that these things depend more on the nature of the tumour than on any other factor.

This investigation is encouraging, however, in that it also suggests that within the limits imposed on us by the character of the tumours which our patients develop, much can be done to assist in controlling the spread. Irradiation reduces the risks of recurrence in the skin and parasternal nodes, and may control the spread seen when metastases have occurred—particularly in skin and in some bones—sometimes for many years.

TABLE III.—*Recurrences in Scar Area.*

A table to show the proportions of first recurrences which involved the scar area for 2 groups of patients :

(*a*) Those who had post-operative H.V.T. to scar area.

(*b*) Those who had no post-operative H.V.T., or at least not to scar area.

(N.B.—Patients who had pre-operative H.V.T. are excluded.)

| | (*a*) H.V.T. to scar. | | | (*b*) No H.V.T. to scar. | | |
|---|---|---|---|---|---|---|
| Year of first recurrence. | Scar area recurrences. | Others. | Total. | Scar area recurrences. | Others. | Total. |
| 1 | 20 | 55 | 75 | 13 | 16 | 29 |
| 2 | 17 | 45 | 62 | 8 | 12 | 20 |
| 3 | 9 | 26 | 35 | 2 | 9 | 11 |
| 4 | 2 | 18 | 20 | 7 | 4 | 11 |
| 5 | 2 | 8 | 10 | 7 | 6 | 13 |
| 6 | 1 | 7 | 8 | 3 | 1 | 4 |
| 7 | 3 | 3 | 6 | 0 | 5 | 5 |
| 8 | 0 | 2 | 2 | 0 | 0 | 0 |
| 9 | 0 | 2 | 2 | 0 | 0 | 0 |
| 10 | 0 | 3 | 3 | 0 | 1 | 1 |
| 11 | 0 | 1 | 1 | 0 | 4 | 4 |
| 5 Year Total | 50 | 152 | 202 | 37 | 47 | 84 |

Percentage first recurrences in first 5 years which involved scar area : (*a*) 24 per cent. (*b*) 44 per cent.

TABLE IV.—*The Effectiveness of Parasternal Irradiation in Preventing Early Parasternal Recurrence.*

| | First treatment including parasternal irradiation. | | First treatment not including parasternal irradiation. | |
|---|---|---|---|---|
| Site of primary growth. | Total first recurrences. | parasternal nodes. | Total first recurrences. | parasternal nodes. |
| Inner half | 21 | 0 | 40 | 7 |
| Outer half | 7 | 0 | 143 | 6 |
| Central | 3 | 0 | 30 | 0 |
| Diffuse | 1 | 0 | 35 | 0 |
| Unspecified | 6 | 0 | 54 | 3 |
| Total | 38 | 0 | 302 | 16 |

I would like to express my thanks to Professor D. W. Smithers for his helpful advice in preparing this paper, and to Mr. P. M. Payne for his invaluable help in the preparation of the tables and figures.

### REFERENCE.

SMITHERS, D. W., RIGBY-JONES, P., GALTON, D. A. G., AND PAYNE, P. M.—(1952) " Cancer of the Breast. A Review." *Brit. J. Radiol.*, Supplement No. 4. London. (British Insitute of Radiology).